# The novel ITPR1 p.Phe2566Ser variant impairs IP_3_R1‐mediated Ca^2+^ release and is associated with ataxia and miosis

**DOI:** 10.1111/joim.70081

**Published:** 2026-02-28

**Authors:** Josephine Wincent, Songbai Zhang, Andrew Nolan, Shigeaki Kanatani, Frida Nordin, Malin Kvarnung, Per Uhlén, Martin Paucar, Ilse Eidhof

**Affiliations:** ^1^ Department of Molecular Medicine and Surgery Karolinska Institute Stockholm Sweden; ^2^ Department of Clinical Genetics and Genomics Karolinska University Hospital Stockholm Sweden; ^3^ Department of Medical Biochemistry and Biophysics Karolinska Institute Stockholm Sweden; ^4^ Division of Eye and Vision St. Erik Eye Hospital Stockholm Sweden; ^5^ Department of Pharmacology and Clinical Neurosciences Umeå University Umeå Sweden; ^6^ Department of Clinical Neuroscience Karolinska Institute Stockholm Sweden; ^7^ Department of Neurology Karolinska University Hospital Stockholm Sweden

**Keywords:** ataxia, calcium signaling, itpr1, miosis

Dear Editor,

Pathogenic variants in the *ITPR1* gene, which encodes the inositol 1,4,5‐trisphosphate receptor type 1 (IP_3_R1) — a critical intracellular calcium (Ca^2+^) release channel — βare known to cause a spectrum of neurological disorders. These include spinocerebellar ataxia types 15 and 29 (SCA15, SCA29), Gillespie syndrome, and pontine/cerebellar hypoplasia. IP_3_R1 is highly expressed in cerebellar Purkinje cells, where it regulates inositol 1,4,5‐trisphosphate (IP_3_)‐evoked endoplasmic reticulum (ER) Ca^2+^ release [1]. Although ataxia is a hallmark of *ITPR1*‐related disease and aniridia or iris hypoplasia is characteristic of Gillespie syndrome, the co‐occurrence of ataxia and miosis has been documented only in two cases [2, 3]. The mechanistic basis of this ultrarare presentation is largely elusive. Here, we report a third family with congenital ataxia and miosis, carrying a novel heterozygous *ITPR1* missense variant.

The index patient was diagnosed with ataxia and miosis in infancy. There was no follow‐up until age 59 when a brain MRI showed mild white matter abnormalities, but no cerebellar atrophy. Upon examination at ages 60 and 62, she displayed mild non‐progressive axial ataxia (SARA score 5.5), mirror movements (MM), broken pursuit, and miosis (Fig. 1a) with slow dark adaptation. OCT showed marked thinning of the iris (Fig. 1b). She did not have pyramidal symptoms or areflexia. The patient's mother, two children, and two grandchildren had ataxia, and four of them had miosis (Fig. 1c, Table S1). Unlike previously reported ataxia–miosis cases [2, 3], none exhibited intellectual disability or dysmorphism (Tables S1 and S2). The iris phenotype of the index, however, closely resembled that described by Chesneau et al. [3], with a thin dilator muscle without iris transillumination, distinguishing it from congenital microcoria [4]. Detailed clinical information can be found in the Supporting Information.

**Fig. 1:**
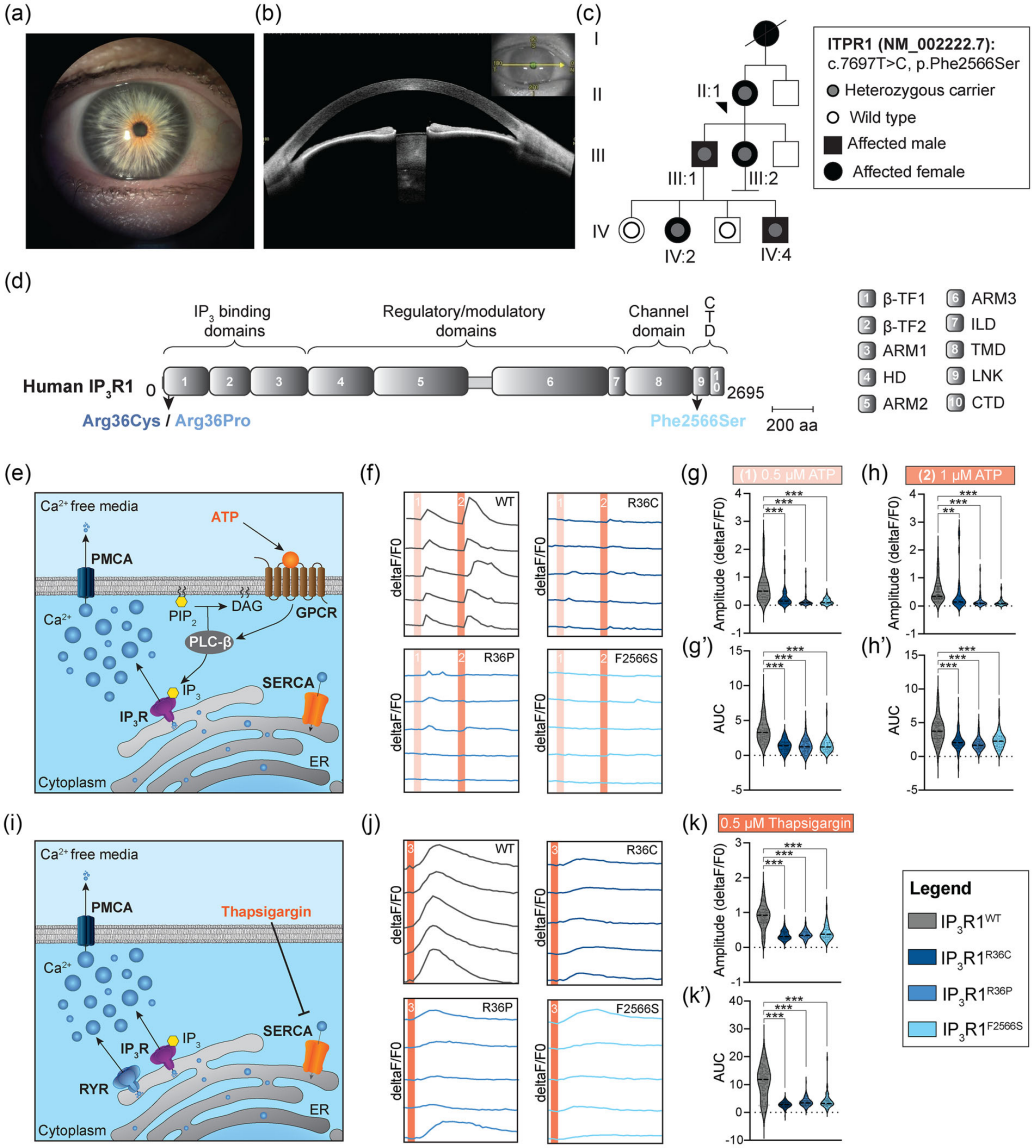
Clinical, genetic, and functional characterization of a novel ITPR1 missense variant underlying ataxia–miosis. (a) Slit‐lamp photograph of the right eye of proband II:1, showing miosis. (b) OCT of the anterior segment of the right eye of proband II:1, revealing a thin iris. (c) Pedigree showing affected individuals in four generations. (d) Each human IP_3_R1 (NP_002213.5) subunit consists of ten protein domains: two b‐trefoil domains (β‐TF1 and β‐TF2), three armadillo selenoid folds (ARM1‐3), an a‐helical domain, an intervening lateral domain (ILD), a transmembrane domain (TMD), a helical linker domain (LNK), and a C‐terminal domain (CTD). (e) Schematic representation of IP_3_R1‐dependent Ca^2+^ release mechanism following ATP stimulation. (f) Representative Ca^2+^ activity trajectories upon 0.5 mM ATP (1) and 1 mM ATP (2) stimulation for the indicated genotypes. (g and h) Quantification of Ca^2+^ peak amplitude (g and h) and Ca^2+^ levels released from the endoplasmic reticulum (ER) (area‐under‐Ca^2+^ curve, g′ and h′) upon ATP stimulation for the indicated concentrations and genotypes. For each condition, the data were normalized to the average response of GFP‐negative cells within the same experimental condition. (i) Schematic representation of mechanism underlying Ca^2+^ release from the ER upon thapsigargin stimulation. (j) Representative Ca^2+^ activity trajectories upon 0.5 mM thapsigargin stimulation (3). (k) Quantification of Ca^2+^ peak amplitude (k) and Ca^2+^ levels released from the ER (area‐under‐Ca^2+^ curve, k′) upon Thapsigargin stimulation for the indicated concentrations and genotypes. **p < 0.01, ***p < 0.001.

Genetic investigation identified the very rare heterozygous *ITPR1* variant c.7697T>C (NM_002222.7, p.Phe2566Ser) with a CADD score of 32, which was reported once in the general population (gnomAD v4.1.0), conserved across species, and co‐segregated with disease. No pathogenic variants were identified in genes associated with MM.

The p.Phe2566Ser variant resided within the IP_3_R1 linker (LNK) domain, distinguishing it from the β‐TF1 suppressor‐domain variants previously associated with ataxia–miosis (Fig. 1d). Using cryo‐EM structures of rat IP_3_R1 [5], we found that the homologous residue of Phe2566 (Phe2621) participates in IP_3_R1 state‐dependent rearrangements (Figs. S2–S4). In the active IP_3_R1, Phe2621 formed many hydrophobic contacts, particularly with His2631 within a Zn^2+^‐binding motif implicated in structural stabilization of the Ca^2+^ pore (Figs. S3 and S4). These interactions were largely lost in inactive or Ca^2+^‐depleted IP_3_R1 channel states. AlphaFold3 [6] modeling of the p.Phe2621Ser substitution predicted disruption of these hydrophobic contacts, altered local folding, and formation of novel interactions, suggesting impaired transmission of regulatory signals that fine‐tune pore opening and stability (Figs. S3–S5).

To test functional consequences, we expressed wild‐type and all known ataxia–miosis IP_3_R1 variants in HEK293T cells. All proteins localized normally to the ER (Fig. S6). Under extracellular Ca^2+^‐free conditions, ATP stimulation revealed significantly reduced IP_3_‐evoked ER Ca^2+^ release in cells expressing ataxia–miosis variant constructs compared with the wild‐type construct (Fig. 1e–h′). Thapsigargin‐evoked ER Ca^2+^ release was likewise diminished, indicating reduced ER Ca^2+^ retention in the absence of ER Ca^2+^ replenishment (Fig. 1i–k′). Control validations verified consistent dye loading and protein construct expression across genetic conditions (Fig. S7), confirming that functional differences were due to the *ITPR1* variants. Together, these results indicate impaired IP_3_R1‐mediated Ca^2+^ signaling and suggest that ataxia–miosis variants cause defects in channel gating, ER Ca^2+^ retention, or both.

Single‐cell transcriptomic analysis demonstrated strong ITPR1 expression in human iris dilator muscle cells, at nearly comparable levels to cerebellar Purkinje cells (Fig S8). This supports a direct, tissue‐specific mechanism for IP_3_R1 in the onset of miosis.

In summary, we provide novel insight into IP_3_R1 ataxia–miosis missense variants and show that they converge on a shared mechanism: impaired IP_3_R1‐mediated Ca^2+^ release and ER Ca^2+^ retention. This distinct pathogenic signature sets these variants apart from *ITPR1* missense variants so far reported in SCA15, SCA29, and Gillespie syndrome. In SCA15, a few functional studies have reported altered IP_3_ binding activity, but maximal IP_3_‐induced Ca^2+^ release appears unaffected, with ER Ca^2+^ levels unexamined [7]. In contrast, SCA29 and Gillespie syndrome missense variants consistently showed reduced IP_3_‐evoked Ca^2+^ release despite normal basal ER Ca^2+^ levels [8, 9]. In Gillespie syndrome, pore‐domain variants appear to diminish channel pore opening [10]. Pore‐proximal variants might thus variably disrupt channel gating, potentially explaining a spectrum of ocular phenotypes — from partial aniridia in Gillespie syndrome to miosis. Further studies should clarify how IP_3_R1 channel variants shape phenotypic variability across *ITPR1*‐related disorders.

## Author contributions

Study conception and design: Josephine Wincent, Songbai Zhang, Andrew Nolan, Per Uhlén, Martin Paucar, and Ilse Eidhof. Investigations: Josephine Wincent, Songbai Zhang, Andrew Nolan, Frida Nordin, Malin Kvarnung, Martin Paucar, and Ilse Eidhof. Methodology: Josephine Wincent, Songbai Zhang, Andrew Nolan, Shigeaki Kanatani, Per Uhlén, Martin Paucar, and Ilse Eidhof. Data analyses: Josephine Wincent, Songbai Zhang, Andrew Nolan, and Ilse Eidhof. Figs. and tables: Josephine Wincent, Songbai Zhang, Andrew Nolan, and Ilse Eidhof. Interpretation of data: all authors. First draft of manuscript: Josephine Wincent, Songbai Zhang, Martin Paucar, and Ilse Eidhof. All authors commented on, read, and approved the final manuscript.

## Conflict of interest statement

The authors declare no conflicts of interest.

## Funding information

Per Uhlén was supported by the Swedish Research Council (2021‐03108), the Swedish Brain Foundation (FO2024‐0057‐TK‐138), the Swedish Cancer Society (22 2454 Pj) and Cancer Research Foundations of Radiumhemmet (221353). Martin Paucar was supprted by Region Stockholm. Ilse Eidhof was supported by a SSMF Grant (PG‐22‐0462).

## Ethics statement

This study was approved by the Swedish Ethical Review Authority (EPN dnr 2016/2538‐32).

## Consent

Informed written consent was obtained from all individuals participating in this study.

## Supporting information




**Table S1**: Phenotype of the affected family members. None of the patients have learning difficulties, dysmorphism, or systemic features. Functional stage (0–6) from Friedreich's ataxia rating scale (FARS). N: normal, NA: not assessed, SARA: scale for the assessment and rating of ataxia, INAS: inventory of non‐ataxia symptoms. *Direction‐changing gaze‐evoked nystagmus. ^a^Age of onset for mirror movements was not possible to determine. ^b^This posturing was dystonic.

**Table S2**: Genetic and clinical information on the current case and previously published cases with miosis and *ITPR1* variants.

**Table S3**: Description of cell types included in Fig. S8.

**Fig. S1**: (a) Slit‐lamp photograph of the right eye of individual III:2, showing miosis. (b) Optical coherence tomography of the anterior segment of the right eye of individual III:2.
**Fig. S2**: (a) AlphaFold3 predicted rat IP_3_R1 protein structure containing the p.Phe2621Ser variant. The amino acid sequence used for the prediction was taken from PDB ID: 7LHE. Color code indicates confidence in the predicted structure. pTM: predicted template modeling score, measuring the accuracy of the entire structure [1]. PLDDT: predicted local distance difference test, providing a per‐atom confidence estimate [1]. (b) ChimeraX overlay of rat tetrameric IP_3_R1 protein structures: CIA‐rIP_3_R1 (dark‐grey), Ca‐rIP_3_R1 (light‐grey), Apo‐rIP_3_R1 (white), and AlphaFold3 rIP_3_R1^Phe2621Ser^ (blue) structures. Zoom in shows Phe2566, corresponding to rat Phe2621 (in orange).
**Fig. S3: Structural impact of the ataxia–miosis variant p.Phe2566Ser on IP_3_R1 Ca^2+^ channel function**. (a) Human ITPR1 (NP_002213.5) and rat ITPR1 (PDB:7LHE) are highly similar on protein level. (b) Amino acid interactions of Phe2621 (in orange) or Ser2621 (in orange) in the indicated structures. (c) Visualization of interaction of Phe2621 or ataxia–miosis residue Ser2621 with the IP_3_R1 C2H2‐like Zn^2+^ finger domain. (d) Heatmap of the predicted number of interactions of the ataxia–miosis residue or the C2H2‐like Zn^2+^ finger domain residues (vertical axis) across rIP_3_R1 protein structures (horizontal axis), as determined by ChimeraX.
**Fig. S4: Predictive structural and molecular interaction analysis of the rIP_3_R1^Phe2621Ser^ variant on the rIP_3_R1 C2H2‐like zinc finger domain**. (a) Visualization of Phe2621 (in orange) or Ser2621 (in orange) interactions with His2631 in indicated protein structures. (b) Heatmap of the predicted interaction frequencies between the indicated amino acids and/or molecules across rIP_3_R1 protein structures, as determined by ChimeraX. The horizontal axis represents the ataxia–miosis‐associated residue or Zn^2+^ finger domain residues. The vertical axis displays amino acids and molecules predicted to interact with either the ataxia–miosis residue or the Zn^2+^ finger domain residues. Hierarchical clustering was applied to both axes using one minus Pearson correlation as the distance metric and the average linkage method.
**Fig. S5: Predicted structural impact of ataxia–miosis variant on Ca^2+^ conduction at IP_3_R1 residue Phe2586**. (a) ChimeraX overlay of rat tetrameric IP_3_R1 protein structures: CIA‐rIP_3_R1 (dark‐grey), Ca‐rIP_3_R1 (light‐grey), Apo‐rIP_3_R1 (white), and AlphaFold3 rIP_3_R1^F2621S^ (blue). (b) ChimeraX measurements of the tetrameric IP_3_R1 Ca^2+^ pore at Phe2586 under different modes of IP_3_R1 activation. In the closed state, F2586 constricts the rIP_3_R1 Ca^2+^ pore; however, upon IP_3_ binding, its side chain rotates away from the pore axis, thereby expanding the channel and permitting Ca^2+^ permeation. The predicted pore opening of rIP_3_R1^F2621S^ resembles that of active CIA‐rIP_3_R1, but not that of inactive Ca‐rIP_3_R1 or Ca^2+^‐depleted Apo‐rIP_3_R1. This implies that the rIP_3_R1 Phe2621Ser variant may promote aberrant Ca^2+^ leak by stabilizing this permeable state.
**Fig. S6**: IP_3_R1‐EGFP variants colocalize with the ER marker KDEL (in red) in HEK293T cells. Nuclei were stained with DAPI.
**Fig. S7**: (a) Representative images of RHOD4‐AM‐loaded cells for the indicated genetic conditions. EGFP‐positive cells correspond to those transfected with the specified IP_3_R1 constructs, whereas EGFP‐negative cells represent non‐transfected cells in the same wells. (b) Baseline Rhod4‐AM fluorescence in EGFP‐negative (non‐transfected) cells did not differ significantly across wells exposed to different transfections, indicating consistent dye loading. (c) Thapsigargin‐induced Ca^2+^ peak amplitudes in EGFP‐negative cells were comparable across all transfected conditions, suggesting no inter‐well variability in the response of non‐transfected cells. (d) Quantification of EGFP fluorescence intensity (pixel values) of transfected cells during live Ca^2+^ imaging for the indicated conditions. *p < 0.05. (e) Linear regression analysis showing the lack of correlation between thapsigargin‐induced Ca^2+^ peak amplitude and EGFP fluorescence intensity in cells expressing different IP_3_R1 constructs across different transfected genetic conditions.
**Fig. S8: ITPR1 is highly expressed in human eye muscle cells and cerebellar Purkinje cells**. (a) T‐SNE plot displaying single‐cell clustering of eye cell types, revealing six major clusters: choroid (red), cornea (blue), iris (green), retina (purple), retinal pigment epithelial cell (RPE, orange), and sclera (brown). (a′) T‐SNE plot as in panel (A), now highlighting the single‐cell expression of ITPR1 across the human eye, demonstrating differential expression patterns among the clusters. (a″) Violin plot depicting normalized ITPR1 expression levels within each eye cell cluster defined in panels (a) and (a′). (b) Scaled ITPR1 expression in cerebellar brain cell types, compared to eye cell types identified in panel A that show high levels of ITPR1 expression. Efferent neurons and brainstem motor neurons from the brain were additionally included in the analysis [2]. Detailed descriptions of all cell types are provided in Table S3.

**Supporting File 1**: joim70081‐sup‐0005‐SuppMat.docx.

**Supporting File 2**: joim70081‐sup‐0006‐SuppMat.docx.

## Data Availability

Raw data are available upon reasonable request to the corresponding authors.
