# Supplementary material for: The novel ITPR1 p.Phe2566Ser variant impairs IP3R1‐mediated Ca2+ release and is associated with ataxia and miosis
Source: J Intern Med. 2026 Feb 28;299(5):643–8. doi: 10.1111/joim.70081 (PMC13061096; doi:10.1111/joim.70081)
Supplement: Supplementary file 1 — Table S1: Phenotype of the affected family members. None of the patients have learning difficulties, dysmorphism, or systemic features. Functional stage (0–6) from Friedreich's ataxia rating scale (FARS). N: normal, NA: not assessed, SARA: scale for the assessment and rating of ataxia, INAS: inventory of non‐ataxia symptoms. *Direction‐changing gaze‐evoked nystagmus. aAge of onset for mirror movements was not possible to determine. bThis posturing was dystonic. [file JOIM-299-643-s004.docx]

**Supplementary table 1:** Phenotype of the affected family members. None of the patients has learning difficulties, dysmorphism or systemic features. Functional stage (0-6) from the Friedreich’s ataxia rating scale (FARS). N: Normal, NA: Not assessed, SARA: Scale for the assessment and rating of ataxia, INAS: inventory of non-ataxia symptoms. *Direction changing gaze-evoked nystagmus. ^a^Age of onset for mirror movements was not possible to determine. ^b^This posturing was dystonic.

| **Patient** | **II:2** | **III:2** | **III:1** | **IV:2** | **IV:4** |
| --- | --- | --- | --- | --- | --- |
| **Age of onset/current age** | Congenital /62 | Congenital /41 | Congenital /43 | Congenital /13 | Congenital/7 |
| **Ataxia** | Yes | Yes | Yes | Yes | Yes |
| **SARA at latest exam (age)** | 5.5 (62) | 6 (40) | 4.5 (42) | 7 | 10 (6) |
| **INAS at last exam** | 0 | 0 | 0 | 0 | 1 |
| **Other neurological features** | Mirror movements (in hands and feet)^a^ | No | No | No | Mild posturing of the feet^b^ |
| **Neurography** | N | NA | NA | NA | NA |
| **Functional stage** | 2 | 2 | 2 | 2 | 2 |
| **Iris abnormalities** | Miosis, thin dilator muscle | Miosis | Miosis | Miosis | Normal |
| **Iris transillumination** | No | No | NA | NA | NA |
| **Eye movements** | Broken smooth pursuit | Broken smooth pursuit | Broken smooth pursuit Hypometric saccades | Broken smooth pursuit | Broken smooth pursuit |
