# Supplementary material for: The novel ITPR1 p.Phe2566Ser variant impairs IP3R1‐mediated Ca2+ release and is associated with ataxia and miosis
Source: J Intern Med. 2026 Feb 28;299(5):643–8. doi: 10.1111/joim.70081 (PMC13061096; doi:10.1111/joim.70081)
Supplement: Supplementary file 3 — Table S3: Description of cell types included in Fig. S8. [file JOIM-299-643-s002.docx]

Table S2: Description of cell-types included in Figure 4.

| **Brain** | | |
| --- | --- | --- |
| **Celltype** | **Ontology ID** | **Description** |
| Purkinje cell | [CL:0000121](https://www.ebi.ac.uk/ols4/ontologies/cl/classes/http%253A%252F%252Fpurl.obolibrary.org%252Fobo%252FCL_0000121) | Purkinje cells are large, uniquely structured inhibitory neurons in the cerebellar cortex that serve as the sole output from the cortex to the cerebellar nuclei. Characterized by their extensive dendritic arbors, they integrate vast excitatory input from parallel and climbing fibers while sending inhibitory signals via a single axon. |
| Cerebellar granule cell precursor | [CL:0002362](https://www.ebi.ac.uk/ols4/ontologies/cl/classes/http%253A%252F%252Fpurl.obolibrary.org%252Fobo%252FCL_0002362) | Granule cell precursors (GCPs) are neuronal progenitor cells essential for generating granule cells, the most abundant neurons in the cerebellum and brain. They play a critical role in the development of the cerebellum and dentate gyrus of the hippocampus. |
| Cerebellar granule cell | [CL:0001031](https://www.ebi.ac.uk/ols4/ontologies/cl/classes/http%253A%252F%252Fpurl.obolibrary.org%252Fobo%252FCL_0001031) | Cerebellar granule cells are the most numerous neurons in the mammalian brain, located in the granular layer of the cerebellum. Despite their small size, they are crucial for coordinating and fine-tuning motor functions through their role in cerebellar circuitry. |
| Stellate neuron | [CL:0000122](https://www.ebi.ac.uk/ols4/ontologies/cl/classes/http%253A%252F%252Fpurl.obolibrary.org%252Fobo%252FCL_0000122) | Stellate neurons are star-shaped interneurons primarily located in the cerebral cortex and cerebellum, distinguished by their radiating dendrites. Their unique morphology supports vital roles in neural communication within mammalian brain circuits. |
| Bergmann glial cell | [CL:0000644](https://www.ebi.ac.uk/ols4/ontologies/cl/classes/http%253A%252F%252Fpurl.obolibrary.org%252Fobo%252FCL_0000644) | Bergmann glial cells are specialized astrocytes in the cerebellum's Purkinje cell layer, characterized by long fibrous processes that extend through the molecular layer alongside Purkinje cell dendrites. They play a crucial role in maintaining cerebellar structure and supporting neuronal function. |
| Efferent neuron | [CL:0000527](https://www.ebi.ac.uk/ols4/ontologies/cl/classes/http%253A%252F%252Fpurl.obolibrary.org%252Fobo%252FCL_0000527) | Efferent neurons, including motor neurons, transmit signals from the brain and spinal cord to muscles and glands, triggering actions like muscle contractions or glandular secretions. They serve as the final link in the neural pathway that enables voluntary and automatic bodily responses. |
| Brainstem motor neuron | [CL:2000047](https://www.ebi.ac.uk/ols4/ontologies/cl/classes/http%253A%252F%252Fpurl.obolibrary.org%252Fobo%252FCL_2000047) | Brainstem motor neurons are specialized nerve cells in the brainstem that play a vital role in coordinating movement by relaying motor signals from the brain to peripheral nerves and muscles. Positioned at the junction between the brain and spinal cord, they serve as essential mediators in the motor system. |
| **Eye** | | |
| **Celltype** | **Ontology ID** | **Description** |
| Ciliary muscle cell | [CL:1000443](https://www.ebi.ac.uk/ols4/ontologies/cl/classes/http%253A%252F%252Fpurl.obolibrary.org%252Fobo%252FCL_1000443) | Ciliary muscle cells are smooth, elongated contractile cells forming the ciliary muscle around the iris in vertebrate eyes. As part of a multinucleated syncytium, they enable functions like lens accommodation without the striations seen in skeletal or cardiac muscle. |
| Muscle cell | [CL:0000187](https://www.ebi.ac.uk/ols4/ontologies/cl/classes/http%253A%252F%252Fpurl.obolibrary.org%252Fobo%252FCL_0000187) | Muscle cells, or myocytes, are specialized contractile cells essential for generating movement and force. They support a range of functions, from voluntary motions to involuntary actions. |
| Smooth muscle cell | [CL:0000192](https://www.ebi.ac.uk/ols4/ontologies/cl/classes/http%253A%252F%252Fpurl.obolibrary.org%252Fobo%252FCL_0000192) | Smooth muscle cells are specialized, involuntary muscle cells found in the walls of hollow organs like blood vessels. They are regulated by the autonomic nervous system and play a vital role in internal bodily functions. |
| Contractile cell | [CL:0000183](https://www.ebi.ac.uk/ols4/ontologies/cl/classes/http%253A%252F%252Fpurl.obolibrary.org%252Fobo%252FCL_0000183) | Contractile cells are specialized for shape change or contraction in response to stimuli, enabling essential physiological functions. |
| Smooth muscle cell of sphincter of pupil | [CL:0002243](https://www.ebi.ac.uk/ols4/ontologies/cl/classes/http%253A%252F%252Fpurl.obolibrary.org%252Fobo%252FCL_0002243) | The smooth muscle cell of sphincter of pupil is a specialized cell type typically found within the eye, more specifically within the sphincter muscle of the iris. |
| Corneal endothelial cell | [CL:0000132](https://www.ebi.ac.uk/ols4/ontologies/cl/classes/http%253A%252F%252Fpurl.obolibrary.org%252Fobo%252FCL_0000132) | Corneal endothelial cells form the innermost layer of the cornea and are essential for maintaining its transparency. They regulate fluid balance by actively pumping water out of the corneal stroma, preventing swelling and preserving clear vision. |
| Melanocyte | [CL:0000148](https://www.ebi.ac.uk/ols4/ontologies/cl/classes/http%253A%252F%252Fpurl.obolibrary.org%252Fobo%252FCL_0000148) | Melanocytes are pigment-producing cells primarily found in the skin, hair, and eyes, as well as other body tissues. They synthesize melanin, which not only determines coloration but also provides protection against harmful ultraviolet radiation. |
| B cell | [CL:0000236](https://www.ebi.ac.uk/ols4/ontologies/cl/classes/http%253A%252F%252Fpurl.obolibrary.org%252Fobo%252FCL_0000236) | B cells, or B lymphocytes, are white blood cells formed in the bone marrow that play a vital role in both adaptive and innate immunity. They produce antibodies that identify and neutralize pathogens like viruses and bacteria. |
| Retinal pigment epithelial cell | [CL:0002586](https://www.ebi.ac.uk/ols4/ontologies/cl/classes/http%253A%252F%252Fpurl.obolibrary.org%252Fobo%252FCL_0002586) | Retinal pigment epithelial (RPE) cells form a pigmented monolayer between the retina and choroid, crucial for supporting photoreceptor health and visual function. They perform key metabolic and transport roles while absorbing excess light to enhance visual clarity and prevent scattering. |
| Interneuron | [CL:0000099](https://www.ebi.ac.uk/ols4/ontologies/cl/classes/http%253A%252F%252Fpurl.obolibrary.org%252Fobo%252FCL_0000099) | Interneurons are a vast class of neurons, that connect sensory and motor neurons to facilitate communication within neural circuits. They play a central role in processing sensory input, generating motor responses, and coordinating reflexes, comprising about 99% of all neurons in the body. |
