# Supplementary material for: The novel ITPR1 p.Phe2566Ser variant impairs IP3R1‐mediated Ca2+ release and is associated with ataxia and miosis
Source: J Intern Med. 2026 Feb 28;299(5):643–8. doi: 10.1111/joim.70081 (PMC13061096; doi:10.1111/joim.70081)
Supplement: Supplementary file 4 — Fig. S1: (a) Slit‐lamp photograph of the right eye of individual III:2, showing miosis. (b) Optical coherence tomography of the anterior segment of the right eye of individual III:2. Fig. S2: (a) AlphaFold3 predicted rat IP3R1 protein structure containing the p.Phe2621Ser variant. The amino acid sequence used for the prediction was taken from PDB ID: 7LHE. Color code indicates confidence in the predicted structure. pTM: predicted template modeling score, measuring the accuracy of the entire structure [1]. PLDDT: predicted local distance difference test, providing a per‐atom confidence estimate [1]. (b) ChimeraX overlay of rat tetrameric IP3R1 protein structures: CIA‐rIP3R1 (dark‐grey), Ca‐rIP3R1 (light‐grey), Apo‐rIP3R1 (white), and AlphaFold3 rIP3R1Phe2621Ser (blue) structures. Zoom in shows Phe2566, corresponding to rat Phe2621 (in orange). Fig. S3: Structural impact of the ataxia–miosis variant p.Phe2566Ser on IP3R1 Ca2+ channel function. (a) Human ITPR1 (NP_002213.5) and rat ITPR1 (PDB:7LHE) are highly similar on protein level. (b) Amino acid interactions of Phe2621 (in orange) or Ser2621 (in orange) in the indicated structures. (c) Visualization of interaction of Phe2621 or ataxia–miosis residue Ser2621 with the IP3R1 C2H2‐like Zn2+ finger domain. (d) Heatmap of the predicted number of interactions of the ataxia–miosis residue or the C2H2‐like Zn2+ finger domain residues (vertical axis) across rIP3R1 protein structures (horizontal axis), as determined by ChimeraX. Fig. S4: Predictive structural and molecular interaction analysis of the rIP3R1Phe2621Ser variant on the rIP3R1 C2H2‐like zinc finger domain. (a) Visualization of Phe2621 (in orange) or Ser2621 (in orange) interactions with His2631 in indicated protein structures. (b) Heatmap of the predicted interaction frequencies between the indicated amino acids and/or molecules across rIP3R1 protein structures, as determined by ChimeraX. The horizontal axis represents the ataxia–miosis‐associated residue or Zn2+ [file JOIM-299-643-s003.docx]

**Supplemental figures 1-8**

**
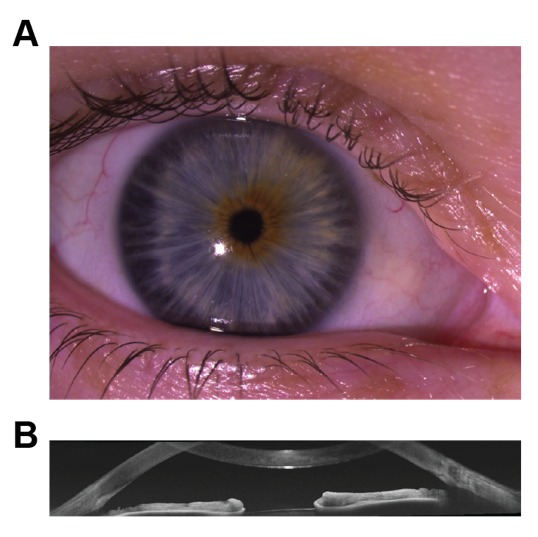
**

**Figure S1: A.** Slit-lamp and pentatax photograph of the right eye of individual III:2, showing miosis. **B.** Optical Coherence Tomography of the anterior segment of the right eye of individual III:2.

**
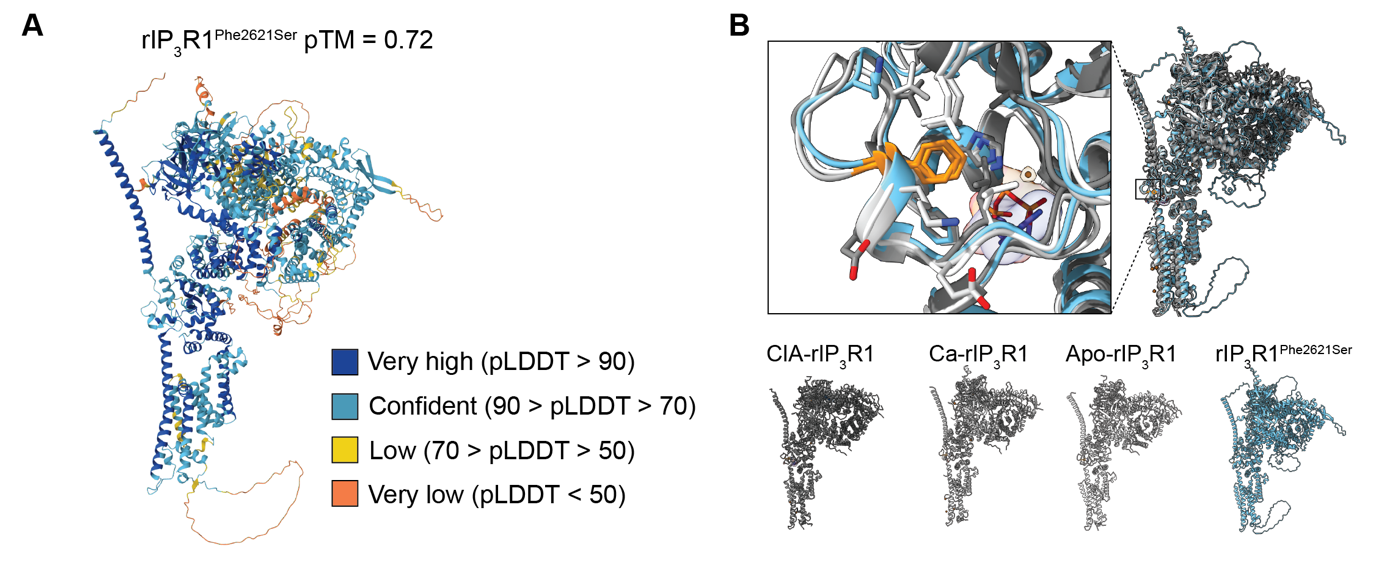
**

**Figure S2: A.** Alphafold3 predicted rat IP_3_R1 protein structure containing the p.Phe2621Ser variant. The amino acid sequence used for the prediction was taken from PDB ID: 7LHE. Color code indicates confidence in the predicted structure. pTM: predicted template modeling score, measuring the accuracy of the entire structure^1^. PLDDT: predicted local distance difference test, providing a per-atom confidence estimate^1^. **B.** ChimeraX overlay of rat tetrameric IP_3_R1 protein structures: CIA-rIP_3_R1 (dark-grey), Ca-rIP_3_R1 (light-grey), Apo-rIP_3_R1 (white) and AlphaFold3 rIP_3_R1^Phe2621Ser^ (blue) structures. Zoom in shows Phe2566, corresponding to rat Phe2621 (in orange).


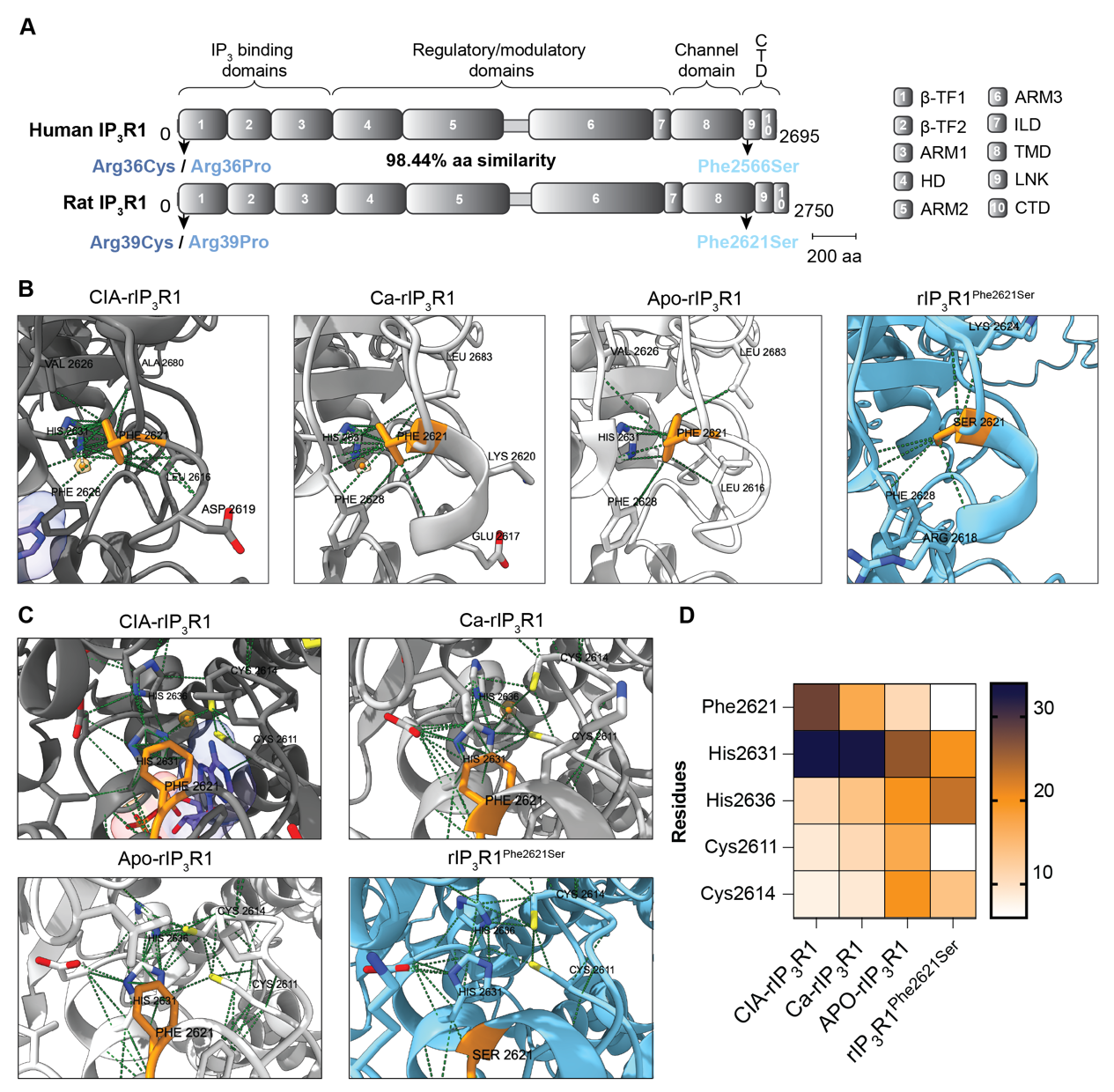


## **Figure S3: Structural impact of the ataxia-miosis variant p.Phe2566Ser on IP_3_R1 Ca^2+^ channel function.** **A.** Human ITPR1 (NP_002213.5) and rat ITPR1 (PDB:7LHE) are highly similar on protein level. **B.** Aminoacid interactions of Phe2621 (in orange) or Ser2621 (in orange) in the indicated structures. **C.** Visualization of interaction of Phe2621 or ataxia-miosis residue Ser2621 with the IP_3_R1 C2H2-like Zn^2+^ finger domain. **D.** Heatmap of the predicted number of interactions of the ataxia-miosis residue or the C2H2-like Zn^2+^ finger domain residues (vertical axis) across rIP_3_R1 protein structures (horizontal axis), as determined by ChimeraX.


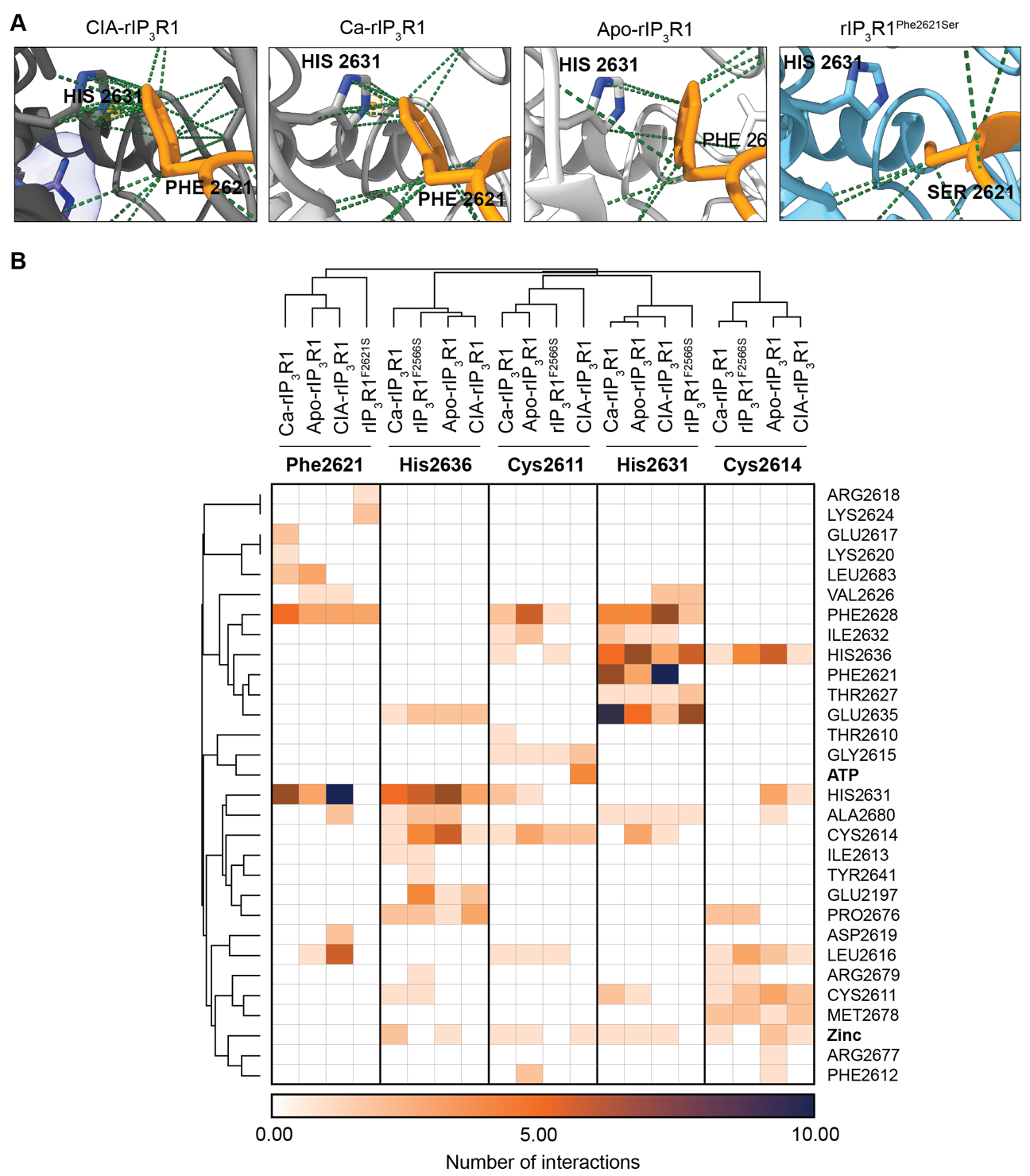


**Figure S4: Predictive structural and molecular interaction analysis of the rIP_3_R1^Phe2621Ser^ variant on the rIP_3_R1 C2H2-Like Zinc Finger Domain.** **A.** Visualization of Phe2621 (in orange) or Ser2621 (in orange) interactions with His2631 in indicated protein structures. **B.** Heatmap of the predicted interaction frequencies between the indicated amino acids and/or molecules across rIP_3_R1 protein structures, as determined by ChimeraX. The horizontal axis represents the ataxia-miosis-associated residue or Zn^2+^ finger domain residues. The vertical axis displays amino acids and molecules predicted to interact with either the ataxia-miosis residue or the Zn^2+^ finger domain residues. Hierarchical clustering was applied to both axes using one minus Pearson correlation as the distance metric and the average linkage method.

**
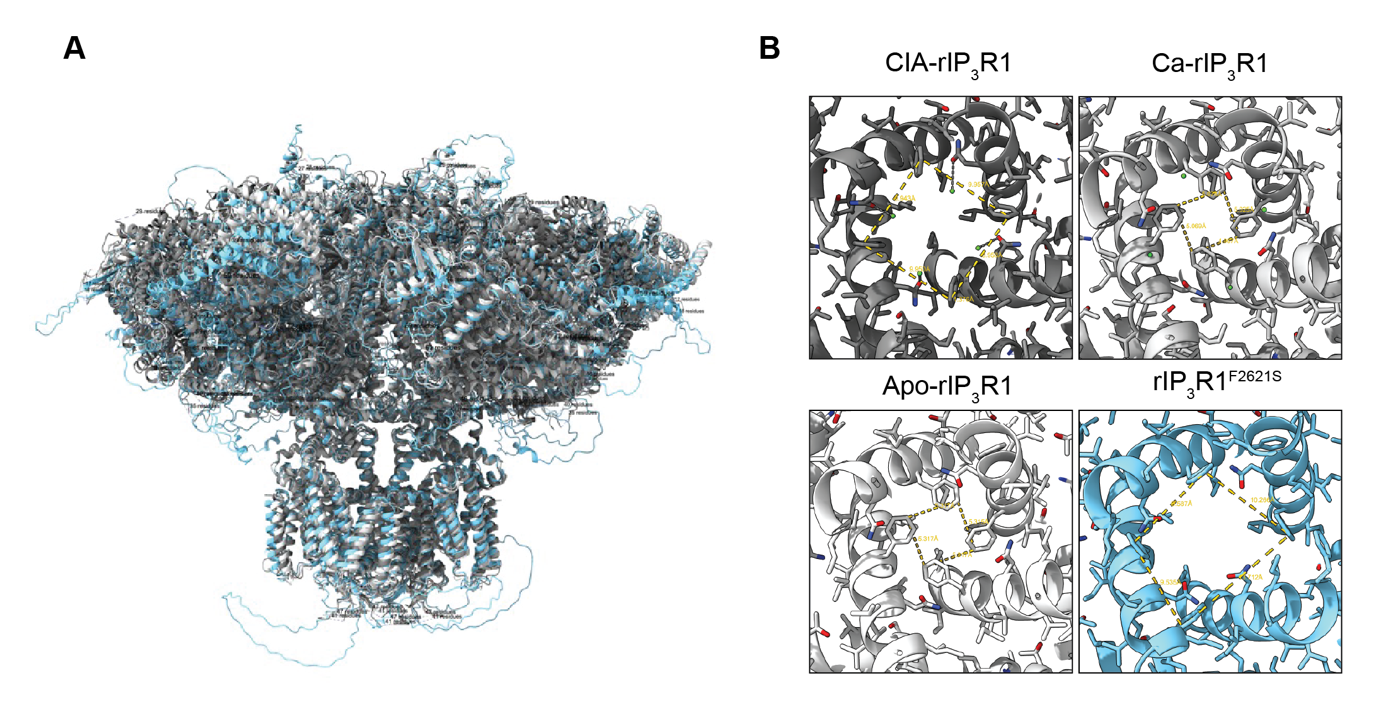
**

**Figure S5: Predicted structural impact of ataxia-miosis variant on Ca^2+^ conduction at IP_3_R1 residue Phe2586. A.** ChimeraX overlay of rat tetrameric IP_3_R1 protein structures: CIA-rIP_3_R1 (dark-grey), Ca-rIP_3_R1 (light-grey), Apo-rIP_3_R1 (white) and AlphaFold3 rIP_3_R1^F2621S^ (blue). **B.** ChimeraX measurements of the tetrameric IP_3_R1 Ca^2+^ pore at Phe2586 under different modes of IP_3_R1 activation. In the closed state, F2586 constricts the rIP_3_R1 Ca²⁺ pore; however, upon IP₃ binding, its side chain rotates away from the pore axis, thereby expanding the channel and permitting Ca²⁺ permeation. The predicted pore opening of rIP_3_R1^F2621S^ resembles that of active CIA-rIP_3_R1, but not that of inactive Ca-rIP_3_R1 or Ca^2+^ depleted Apo-rIP_3_R1. This implies that the rIP_3_R1 Phe2621Ser variant may promote aberrant Ca²⁺ leak by stabilizing this permeable state.


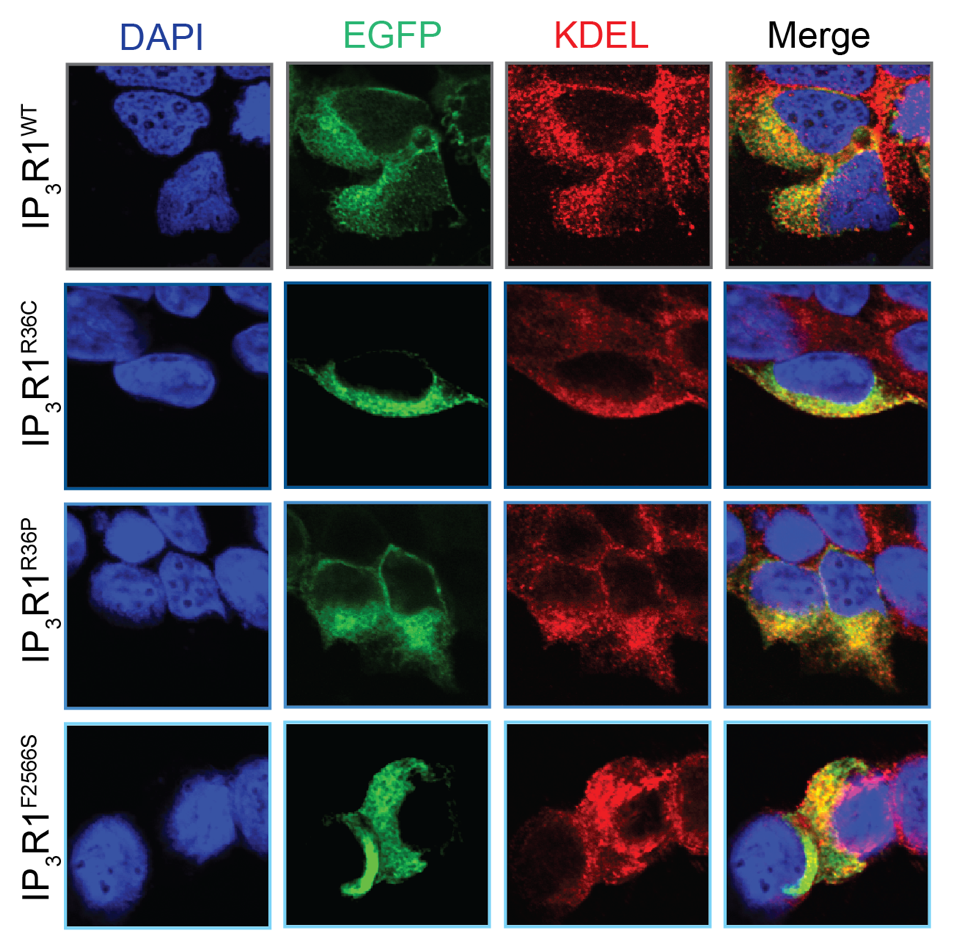


**Figure S6:** IP_3_R1-EGFP variants colocalize with the ER marker KDEL (in red) in HEK293T cells. Nuclei were stained with DAPI.

**
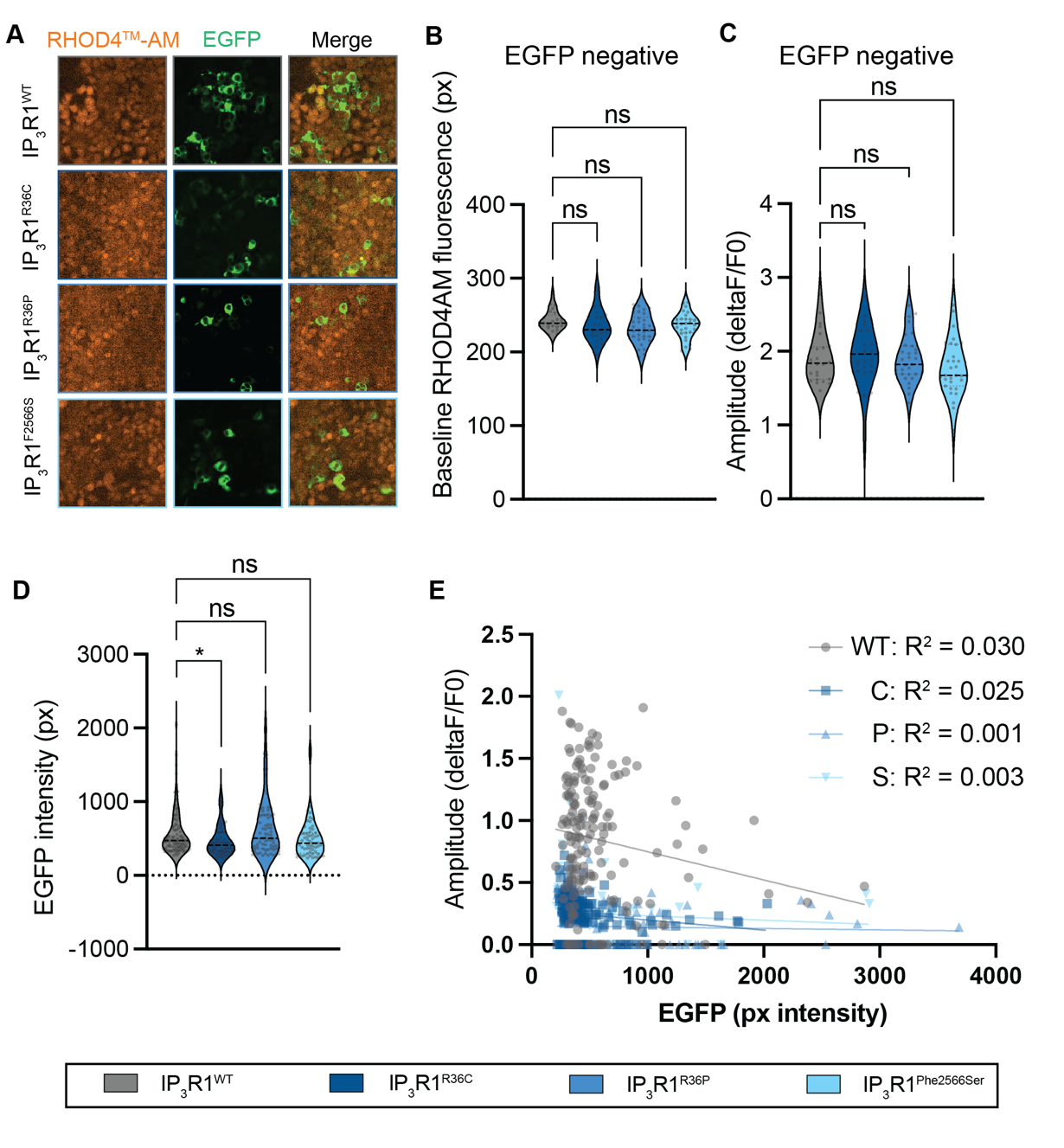
**

**Figure S7: A.** Representative images of RHOD4^TM^-AM loaded cells for the indicated genetic conditions. EGFP-positive cells correspond to those transfected with the specified IP_3_R1 constructs, whereas EGFP-negative cells represent non-transfected cells in the same wells. **B.** Baseline Rhod4-AM fluorescence in EGFP-negative (non-transfected) cells did not differ significantly across wells exposed to different transfections, indicating consistent dye loading. **C.** Thapsigargin-induced Ca^2+^ peak amplitudes in EGFP-negative cells were comparable across all transfected conditions, suggesting no inter-well variability in the response of non-transfected cells. **D.** Quantification of EGFP fluorescence intensity (pixel values) of transfected cells during live Ca^2+^ imaging for the indicated conditions. *p < 0.05. **E.** Linear regression analysis showing the lack of correlation between thapsigargin-induced Ca^2+^ peak amplitude and EGFP fluorescence intensity in cells expressing different IP_3_R1 constructs across different transfected genetic conditions.


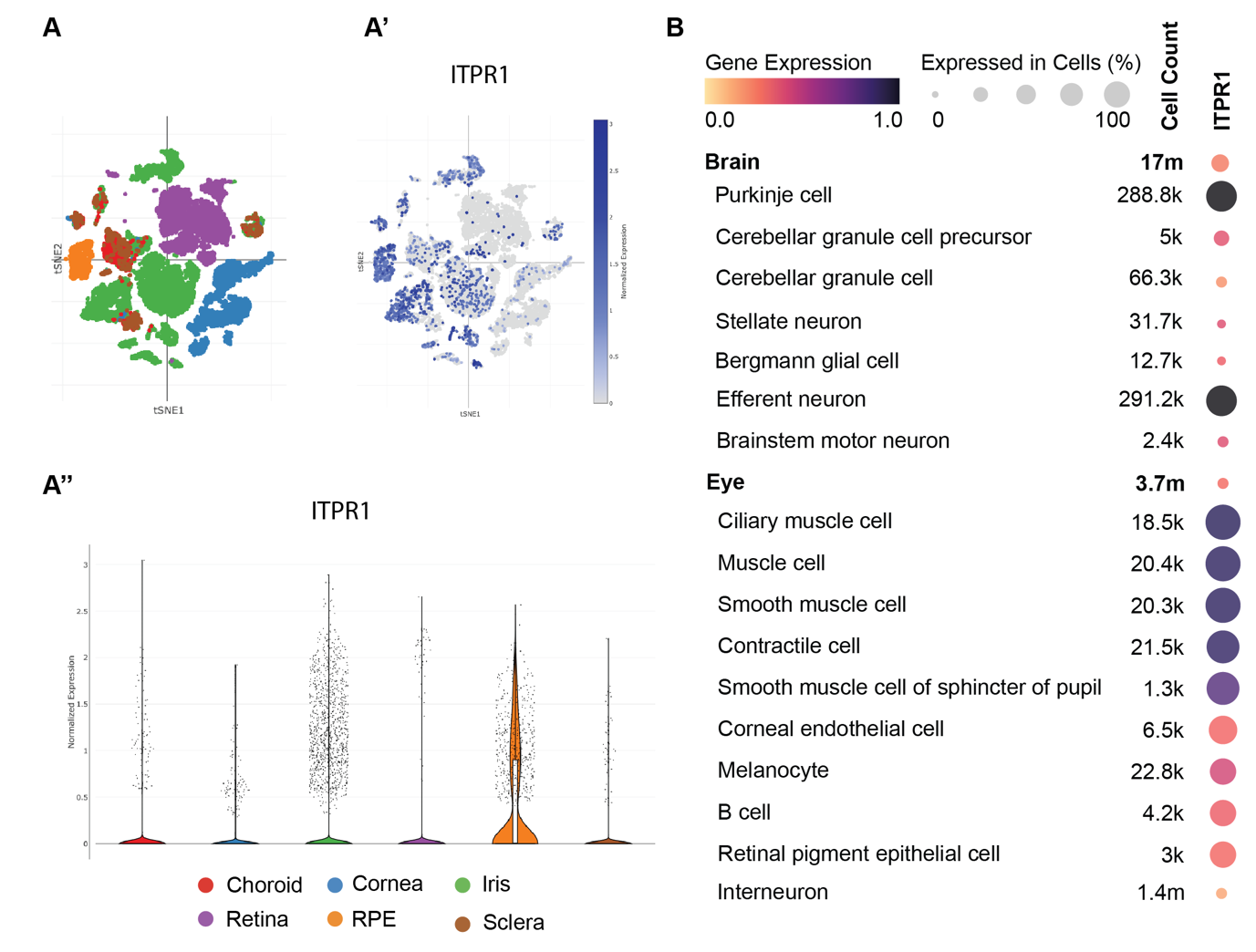


**Figure S8: ITPR1 is highly expressed in human eye muscle cells and cerebellar Purkinje cells. A.** T-SNE plot displaying single cell clustering of eye cell types, revealing 6 major clusters: Choroid (red), Cornea (blue), Iris (green), Retina (purple), Retinal pigment epithelial cell (RPE, orange) and Sclera (brown). **A’.** T-SNE plot as in A, now highlighting the single-cell expression of ITPR1 across the human eye, demonstrating differential expression patterns among the clusters. **A’’.** Violin plot depicting normalized ITPR1 expression levels within each eye cell cluster defined in panels A and A’. **B.** Scaled ITPR1 expression in cerebellar brain cell types, compared to eye cell types identified in panel A that show high levels of ITPR1 expression. Efferent neurons and brainstem motor neurons from the brain were additionally included in the analysis^2^. Detailed descriptions of all cell types are provided in Table S3.

**References**

1 Abramson, J. *et al.* Accurate structure prediction of biomolecular interactions with AlphaFold 3. *Nature* **630**, 493-500 (2024). <https://doi.org:10.1038/s41586-024-07487-w>

2 CZI Single-Cell Biology Program, S. A. *et al.* A single-cell data platform for scalable exploration, analysis and modeling of aggregated data. *BioRxiv* (2023). <https://doi.org:https://doi.org/10.1101/2023.10.30.563174>
