# Supplementary material for: The novel ITPR1 p.Phe2566Ser variant impairs IP3R1‐mediated Ca2+ release and is associated with ataxia and miosis
Source: J Intern Med. 2026 Feb 28;299(5):643–8. doi: 10.1111/joim.70081 (PMC13061096; doi:10.1111/joim.70081)
Supplement: Supplementary file 5 — Supporting File 1: joim70081‐sup‐0005‐SuppMat.docx. [file JOIM-299-643-s001.docx]

**Supplementary Clinical Data**

The pedigree is displayed in **Figure 1C**.

Patient 1, the index case in this family, is a 62-year old female (II:1). Other than ataxia, mirror movements (MM) were also found upon examination. These MM were present as for as long as the patient could remember. The patient reported difficulty seeing when initially entering a dark room, and some difficulty following moving objects, otherwise no visual complaints. She had good best-corrected visual acuity (BCVA) of 1,2 and normal intraocular pressure in both eyes. Slit lamp examination revealed an interesting pigmentation of the iris, with darker blue in the periphery and lighter blue centrally (**Fig. 1A**). No iris transillumination. The pupils of both eyes were about 2 mm in the slit lamp. Normal, even constriction of the pupil to light. After installment of 0,5% Tropikamid and Phenylephrine (unknown concentration), the pupils did not dilate. Discrete nuclear sclerosis of the lens. No visual field defects on confrontation. Ocular motility was assessed using video oculography and revealed broken pursuit. Normal fixation, saccades, VOR-suppression and OKN response. No nystagmus was observed. Ocular coherence tomography (OCT) showed normal values of both peripapillary retinal nerve fiber layers (pRNFL) and ganglion cell layers (GCL). OCT of the anterior chamber showed a thin iris, particularly from mid to periphery (**Fig. 1B**).

Patient 2 (III:2), the daughter of patient 1, was a 41-year old female who reported small pupils for as long as she can remember. The only visual complaint was a prolonged time to adjust to dark conditions, which could be up to five minutes. Examination showed a normal BCVA of 1,5 bilaterally and normal intraocular pressures. We observed a bilateral, symmetric miosis (**Fig. S1A**). The pupil size in a brightly lit room was 1 mm and in a dark room was 2 mm. The pupils reacted briskly to light and had no apparent dilation lag. Light-near-dissociation was absent, i e the pupils constricted during convergence. After installment of 0,5% Tropikamid, the pupils dilated to 4 mm. When testing ocular motility, smooth pursuit toward the right had a decreased gain with small catch-up saccades. Range of movement, fixation, saccades, and VOR-suppression were deemed normal. No strabismus was present. Optical Coherence Tomography (OCT) of the pRNFL and GCL of the macula showed normal values (**Fig. S1B**). Slit lamp examination of the eye revealed no abnormal findings.

None of the other ataxia patients in this family than the index case had MM.
