# Supplementary material for: The novel ITPR1 p.Phe2566Ser variant impairs IP3R1‐mediated Ca2+ release and is associated with ataxia and miosis
Source: J Intern Med. 2026 Feb 28;299(5):643–8. doi: 10.1111/joim.70081 (PMC13061096; doi:10.1111/joim.70081)
Supplement: Supplementary file 6 — Supporting File 2: joim70081‐sup‐0006‐SuppMat.docx. [file JOIM-299-643-s005.docx]

**Supplementary methodology**

## **Clinical Investigations**

The medical records for each family member were reviewed. Ocular motility was assessed using an eye tracker (VisualEyes 525) and clinical observation. The iris of the index case (II:1) and her daughter (III:2) were examined with slit-lamp and optical coherence tomography (OCT).

## **Massive parallel sequencing and Sanger sequencing**

Massive parallel sequencing (MPS) of the index case (proband II:2) was performed using a 30× PCR-free paired-end WGS protocol on an Illumina NovaSeq 6000 platform as described previously^1^. A gene panel of 956 genes associated with movement disorders and neuromuscular disease was analyzed. The MPS analysis comprised coding regions, exon-intron boundaries, copy-number variant detection, and analysis for balanced structural variants involving the genes included in the panel. Repeat expansion analysis of relevant genes was also included. The variants were prioritized based on conservation, frequency in internal and public databases, and pattern of inheritance. The ranked variants were then visualized in the Scout analysis platform^2^. Genes associated with mirror movements were also analyzed (*DCC*, *RAD51*, *NTN1*, and *ARHGEF7*). The *ITPR1-*variant was confirmed to segregate with disease in the family by PCR and Sanger sequencing. The Sanger sequencing was performed by standard methods on an ABI 3730 PRISM® DNA Analyzer. Primer sequences available upon request.

## **Structural protein modeling**

The following cryo-EM structures of rat IP_3_R1 (sharing 98.44% identity with human IP_3_R1) were used and investigated using ChimeraX^3^: activated (Ca^2+^, IP_3_ and ATP bound: CIA-rIP_3_R1 (PDB ID: 8EAR)), inhibited (high Ca^2+^ bound: Ca-rIP_3_R1 (PDB ID: 8EAQ)), and Ca^2+^ depleted (Apo-rIP_3_R1 (PDB ID: 7LHE))^4^. To investigate whether the p.Phe2621Ser variant mimics wild-type Phe2621 interactions, we used AlphaFold3^5^ to predict the rIP_3_R1^Phe2621Ser^ protein structure. The predicted model exhibited high confidence, particularly in the channel-gating machinery containing the variant (Fig. S1A). We subsequently superimposed it (Fig. S1B, in blue) onto CIA-rIP_3_R1, Ca-rIP_3_R1 and APO-rIP_3_R1 using ChimeraX^3^ and investigated Ser2621’s orientation and contacts.

## **Plasmid generation**

To construct EGFP-IP_3_R1-WT, the *NheI*-EGFP-IP_3_R1-*XhoI* fragment (restriction enzymes are italicized, same as below) was cut from EGFP-mIP_3_R1-N ^6^ and inserted into CAG-MCS2 ^7^. The site-directed mutants of EGFP-IP_3_R1were generated using Pfu Turbo DNA Polymerase (Cat. 600250, Agilent Technologies, Santa Clara, CA, USA) following the manufactures protocol. To generate EGFP-IP_3_R1-R36C and EGFP-IP_3_R1-R36C, the fragment *KpnI*-EGFP-IP_3_R1/N-*NheI* fragment was cut from EGFP-IP_3_R1-WT and inserted into pcDNA-mRFP-GIT1 ^8^, resulting in the plasmid pcDNA-IP_3_R1/N-EGFP. PCR for IP_3_R1/N-R36C and IP_3_R1/N-R36P was performed using pcDNA-IP3R1/N-EGFP as the template with the following primers (underline indicates mutated nucleic acid, same as below): 5′-GGCTTGGTTGATGACTGTTGTGTTGTACAGC-3′, 5′-CTTGGTTGATGACCCTTGTGTTGTACAGC-3′, respectively. The fragments of *KpnI*-IP_3_R1/N-R36C-EGFP-*NheI* and *KpnI*-IP_3_R1/N-R36P-EGFP-*NheI* were confirmed by sequencing and replaced into EGFP-IP_3_R1-WT, resulting in EGFP-IP_3_R1-R36C and EGFP-IP_3_R1-R36P, respectively. To generate EGFP-IP_3_R1-F2620S, the *EcoRI*-IP_3_R1/C-*XhoI* fragment was cut from GFP-mIP_3_R1-N and inserted into pcDNA-mRFP-GIT1, resulting in the plasmid pcDNA-IP_3_R1/C. PCR for IP_3_R1/C-F2620S was performed using pcDNA-IP_3_R1/C as the template with the following primer: 5′-GGCTTGGAAAGGGACAAGTCTGACAATAAGACTGTCACC-3′. The *EcoRI*-IP_3_R1/C-F2620S-*XhoI* fragments was confirmed by sequencing and replaced into GFP-mIP_3_R1-N, resulting in GFP-mIP_3_R1-F2620S-N. EGFP-IP_3_R1-F2620S was produced by insertion of the *NheI*-GFP-mIP_3_R1-F2620S-*XhoI* fragment from GFP-mIP_3_R1-F2620S-N into CAG-MCS2.

## **Cell culture**

HEK-293T cells were purchased (Sigma-Aldrich, Cat. 12022001-1VL) and maintained in DMEM medium (Gibco, Cat. 31966-021) supplemented with 10% fetal bovine serum (FBS, Invitrogen, Cat. 25149-079) and 1% Antibiotic-Antimycotic (Gibco, Cat. 15240062).

## **Ca^2+^ imaging**

3 ×10^4^ cells per well were seeded in Poly-L-Lysine (Sigma, Cat. P4707) coated 96 Well µ-Plates (Ibidi, Cat. 89626) and transfected the next day using Lipofectamine™ 2000 (Invitrogen, Cat. 11668027) according to manufacturer's protocol. After two days, IP_3_R1 functionality was assessed using Ca^2+^ imaging. In brief, cells were loaded with the Ca^2+^-sensitive fluorescent indicator RHOD4^TM^-AM (10 µM; AAT Bioquest, Cat. ABD-21122) in the presence of 0.04% Pluronic F-127 (ThermoFisher Scientific, Cat. P3000MP) and incubated for 20 min at 37°C in 1x Krebs-Ringer buffer. The buffer was composed of: NaCl (119 mM, Cat. S7653), KCl (2.5 mM, Cat. P5405), NaH_2_PO_4_ monobasic (1 mM, Cat. S3139), CaCl_2_×2H_2_O (2.5 mM, Cat. C3306), MgCl_2_×6H_2_O (1.3 mM, Cat. M2393), HEPES (20 mM, Cat. H4034) and D-Glucose (11 mM, Cat. G8270), with pH adjusted to 7.4 (all from Sigma-Aldrich). Following dye incubation, cells were washed to remove excess dye and incubated for an additional 20 min in 1x KREBS Ringer buffer. Transfected cells were imaged at 37°C using a Nikon CrEST X-Light V3 inverted confocal spinning disk microscope with a 20x/0.8 dry lens (Nikon) in 1x Ca^2+^ free Krebs-Ringer buffer containing 2mM EGTA (Sigma-Aldrich, Cat. E4378), in which CaCl_2_×2H_2_O was omitted. Excitation was assessed at 477 nm and 546 nm for all genotypes (4 x 2 wells) simultaneously, for 15 min at 0.5 Hz. The equipment was controlled with, and imaging data was collected using NIS Elements software (Nikon). After 5 min, cells were stimulated with either 0.5 mM ATP, followed by 1 mM ATP (Sigma-Aldrich, Cat. A9187) or 0.5 mM thapsigargin (ThermoFisher Scientific, Cat. T7459). FIJI, MATLAB (R2021a, MathWorks, USA) and FluoroSNNAP^9^ were used to process and analyze the collected data.

## **Immunocytochemistry**

1 ×10^4^ cells per well were seeded in an 8-well-culture-chamber (Falcon, Cat. 354108). One day later, transfection was performed using 92 ng of cDNA per well with 0.46 ml per well of Lipofectamine™ 2000 Transfection Reagent. After two additional days, the cells were washed once with PBS, treated at 4 ℃ for 10 minutes with Methanol pre-cold to -20 °C, permeabilized with P-buffer (0.1% Triton X-100 + 0.1% Tween in PBS) at room temperature for 10 minutes, and then blocked with B-buffer (5% skim milk in P-buffer) at room temperature for 1 hour. The cells were subsequently incubated with the primary antibody, anti-KDEL (Santa Cruz Biotechnology, Cat. sc-58774, diluted at 1:250 in B-buffer), followed by the secondary antibody, anti-Mouse IgG (H+L) Alexa Fluor™ 555 (Invitrogen, Cat. A-21422, diluted 1:500 in B-buffer). Coverslips were mounted using Vectashield Antifade Mounting Medium with DAPI (Vector Laboratories, Cat. H-1200-10). Fluorescence images were acquired using a Olympus FluoView1000 confocal microscope (Olympus, Tokyo, Japan) and analyzed with Olympus FV10-ASW software.

**Statistical analysis**

The results were plotted in PRISM (GraphPad Prism 9). Statistical analysis of live Ca^2+^ imaging was done with a non-parametric Kruskal-Wallis test in combination with a Dunns multiple comparisons post-test. P-values lower than 0.05 were considered as significant, with * p < 0.05, ** p < 0.01, *** p < 0.001, **** p < 0.0001.

**Single Cell RNA Sequencing analysis**

CZ CELLxGENE Discover^10^ was used to locate relevant human eye and brain single-cell RNA-seq datasets by filtering on the metadata (organism, tissue/organ) and selecting all datasets corresponding to human eye and brain tissues. ITPR1 expression was then queried and scaled across relevant human eye- and cerebellar and brainstem cell types, to allow for comparison in expression levels and specificity. Cell populations of interest were defined using standard metadata categories and interactive cell subsetting. Single-cell gene expression results were interpreted using the dot plot summaries, reflecting both the fraction of expressing cells and average expression per group. For ITPR1 expression in human eye cell populations, single-cell gene expression was visualized in tSNE and violin plots using the platforms’ interactive gene expression views and metadata-driving grouping. Source data was downloaded on 29/01/2025.

**Supplementary references**

1 Magnusson, M. *et al.* Loqusdb: added value of an observations database of local genomic variation. *BMC Bioinformatics* **21**, 273 (2020). <https://doi.org:10.1186/s12859-020-03609-z>

2 Stranneheim, H. *et al.* Integration of whole genome sequencing into a healthcare setting: high diagnostic rates across multiple clinical entities in 3219 rare disease patients. *Genome Med* **13**, 40 (2021). <https://doi.org:10.1186/s13073-021-00855-5>

3 Meng, E. C. *et al.* UCSF ChimeraX: Tools for structure building and analysis. *Protein Sci* **32**, e4792 (2023). <https://doi.org:10.1002/pro.4792>

4 Fan, G. *et al.* Conformational motions and ligand-binding underlying gating and regulation in IP(3)R channel. *Nat Commun* **13**, 6942 (2022). <https://doi.org:10.1038/s41467-022-34574-1>

5 Abramson, J. *et al.* Accurate structure prediction of biomolecular interactions with AlphaFold 3. *Nature* **630**, 493-500 (2024). <https://doi.org:10.1038/s41586-024-07487-w>

6 Nakayama, T. *et al.* The regulatory domain of the inositol 1,4,5-trisphosphate receptor is necessary to keep the channel domain closed: possible physiological significance of specific cleavage by caspase 3. *Biochem J* **377**, 299-307 (2004). <https://doi.org:10.1042/BJ20030599>

7 Kawauchi, T., Chihama, K., Nishimura, Y. V., Nabeshima, Y. & Hoshino, M. MAP1B phosphorylation is differentially regulated by Cdk5/p35, Cdk5/p25, and JNK. *Biochemical and biophysical research communications* **331**, 50-55 (2005). <https://doi.org:10.1016/j.bbrc.2005.03.132>

8 Zhang, S., Hisatsune, C., Matsu-Ura, T. & Mikoshiba, K. G-protein-coupled receptor kinase-interacting proteins inhibit apoptosis by inositol 1,4,5-triphosphate receptor-mediated Ca2+ signal regulation. *The Journal of biological chemistry* **284**, 29158-29169 (2009). <https://doi.org:10.1074/jbc.M109.041509>

9 Patel, T. P., Man, K., Firestein, B. L. & Meaney, D. F. Automated quantification of neuronal networks and single-cell calcium dynamics using calcium imaging. *J Neurosci Methods* **243**, 26-38 (2015). <https://doi.org:10.1016/j.jneumeth.2015.01.020>

10 CZI Single-Cell Biology Program, S. A. *et al.* A single-cell data platform for scalable exploration, analysis and modeling of aggregated data. *BioRxiv* (2023). <https://doi.org:https://doi.org/10.1101/2023.10.30.563174>
